# Assessment of Patient-Centered Outcomes When Treating Palatally Impacted Canines Using Conventional Versus Accelerated Minimally Invasive Corticotomy-Assisted Orthodontic Treatment: A Randomized Controlled Trial

**DOI:** 10.7759/cureus.30392

**Published:** 2022-10-17

**Authors:** Mahran Raheel Mousa, Mohammad Y Hajeer, Ahmad S Burhan, Omar Heshmeh, Khaldoun Darwich

**Affiliations:** 1 Department of Orthodontics, University of Damascus Faculty of Dentistry, Damascus, SYR; 2 Department of Oral and Maxillofacial Surgery, University of Damascus Faculty of Dentistry, Damascus, SYR

**Keywords:** discomfort, mastication, swallowing, patient-reported outcome measures, visual analog scale, impacted canine withdrawal, impacted canine traction, minimally-invasive corticomy-assisted treatment, canine impaction, palatally impacted canine

## Abstract

Objective

This study aimed to investigate whether there were any differences in pain levels, discomfort, and functional impairments when treating palatally impacted canines (PICs) using the conventional treatment method compared to the accelerated minimally invasive corticotomy-assisted method.

Materials and methods

Fifty-two patients (11 males and 41 females) with unilateral PICs were included. The patients were randomly assigned to the conventional traction group (26 patients, mean age of 20.37 ± 2.15 years) or the minimally-invasive corticotomy-assisted group (26 patients, mean age of 20.18 ± 2.18 years). The levels of pain, discomfort, and functional difficulties were assessed using a visual analog scale (VAS) after 24 hours (T1), four days (T2), seven days (T3), 14 days (T4), and 28 days (T5) following the surgical exposure procedure.

Results

There were no statistically significant differences between the two treatment groups for any patient-centered outcome at all assessment times (P>0.01). The levels of pain and discomfort were slightly greater in the conventional group than in the corticotomy-assisted group on the first day after surgical exposure, with no significant difference between the two groups (mean pain: 4.11, P=0.481; mean discomfort: 9.00, P=0.223). Pain and discomfort required seven days to reach low levels and four weeks to reach the lowest levels in both study groups. The levels of swelling, mastication difficulties, swallowing difficulties, limitation in jaw movements and speech changes were mild to moderate on the first postoperative day and the recovery time was four days postoperatively for swallowing difficulties and speech changes. In comparison, the recovery time was seven days for the other three outcomes in both study groups.

Conclusions

After one day of the surgical intervention, either by conventional or corticotomy-assisted methods, the patients reported mild to moderate pain, discomfort, and functional impairments. These disabilities gradually reached low levels during the first and second weeks to reach their lowest levels four weeks postoperatively in both study groups. The similarity between the conventional and the acceleration methods in pain levels and other oral disabilities may make corticotomy-assisted treatment a comfortable and effective method when treating adult patients with PICs. In addition, patient satisfaction with the corticotomy-assisted procedure was high.

## Introduction

The impacted canine is described as the complete or partial failure of a tooth emergence within six months of complete root formation [1]. Impacted upper canines are found in approximately 2% of the general population, occurring more than twice as frequently in women (1.17%) than in men (0.51%), and palatally impaction is four times more common than buccal impaction (60-80%) [2-4]. The mean age of maxillary canine eruption is 10.5 years in girls and 11.5 years in boys [5]. Several factors, such as hard or soft tissue obstructions or eruption pathway deviation, can lead to the failure of the upper canine's eruption [6]. In addition, many factors lead to the displacement of the impacted canine towards the palatal side, such as retained deciduous canines, arch length discrepancy, as well as trauma during the formation of the canines [7]. Surgical exposure and orthodontic traction are the most recommended methods for maxillary-impacted canine treatment [8]. A combination of surgical/orthodontic approaches aims to reposition impacted canines in their place on the dental arch without causing periodontal damage. To achieve this goal, several mechanical traction methods and two basic surgical methods (open and closed) are used depending on the impacted canine location, and the ligation technique used [9,10]. However, most surgical procedures may accompany complications and secondary effects depending on periodontal and bone tissue damage [11].

In the medical literature, several studies evaluated patients' perceptions of pain, discomfort, acceptance, and health-related quality of life associated with different types of orthodontic treatment interventions, such as functional treatments of skeletal class II and III cases [12,13], rapid and slow maxillary expansion [14], lingual orthodontics [15,16], and clear aligners [17]. However, very few studies have been conducted to determine the postoperative complications associated with the treatment of impacted maxillary canines [18-22].

When the open and closed surgical methods were compared, Björksved M et al. [18] found that patients experienced significantly more post-surgery pain and impairment, analyzed from two questionnaires on the first and seventh day after exposure in the open technique group. Gharaibeh TM and Al-Nimri KS [19] found no differences in the perceptions of pain between the two surgical methods for seven days after surgery using a numerical rating scale. Parkin NA et al. [20] found similar results, which were analyzed from a postoperative questionnaire on the 10th day after surgical exposure. Chaushu S et al. [21] found that when using the open-eruption technique, severe pain continued until the seventh day after surgery, and the postoperative recovery was longer and significantly more impaired compared to the closed-eruption group.

Heravi F et al. [22] reported that the mean values of patient-perceived pain, measured by a visual analog scale (VAS), were not different between study groups, miniscrews vs. trans-palatal arch groups, using the open-eruption method in the two compared groups. The results of a recent systematic review by Mousa MR et al. [10] revealed that the pain levels did not differ significantly between trials that used different conventional exposure methods of palatally impacted canines (PICs) in the short-term follow-up period of 1-10 days, while the results of pain incidence were inconsistent between these trials [10].

There is a possibility to accelerate the traction movement of impacted canines [23-25] using surgical and non-surgical accelerating methods. However, when reviewing the available literature, it appears that there are few studies about the acceleration of impacted canines' movement. In addition, the pain, discomfort, and functional impairments related to the acceleration procedures used in these studies have not been evaluated [26].

In the current clinical practice, patients undergoing orthodontic/surgical treatment for impacted canines request more detailed information about potential postoperative pain and discomfort. Accordingly, this randomized controlled trial aimed to assess patients' perceptions of pain, discomfort, and functional impairments immediately after the surgical exposure of PICs treated with the conventional closed-eruption treatment technique versus the minimally invasive, surgically assisted acceleration technique.

## Materials and methods

Study design and registration

This was a randomized controlled study with two parallel groups designed to assess whether there were any differences in pain, discomfort, and functional impairment levels accompanying conventional versus minimally invasive, surgically assisted orthodontic treatment of PICs in adult patients. Patients with impacted upper canines registered at the Department of Orthodontics at Damascus University, Dental School, were examined between September 2018 and December 2021. This research project was approved by the Local Research Ethics Committee of the Damascus University, Dental School (UDDS-491-01012019/SRC-2180). The study was previously registered at ClinicalTrials.gov (ID: NCT03678805) and funded by Damascus University, Postgraduate Research Budget (Ref no: 83004661002DEN).

Sample size calculation

The sample size was calculated using Minitab® Version 17 (Minitab Inc., State College, Pennsylvania, USA). The smallest clinically important difference requiring detection between the two study groups was 1 cm on a VAS with an SD of 1.2 based on a previous study [18]. An independent sample t-test was performed with a significance level of 5% and a power of 80%. This test showed that 24 patients were required in each group. To avoid any potential attrition, two patients were added to each group, and the final number of patients reached 26 in each group.

Patients' recruitment and eligibility criteria

**Figure 1:**
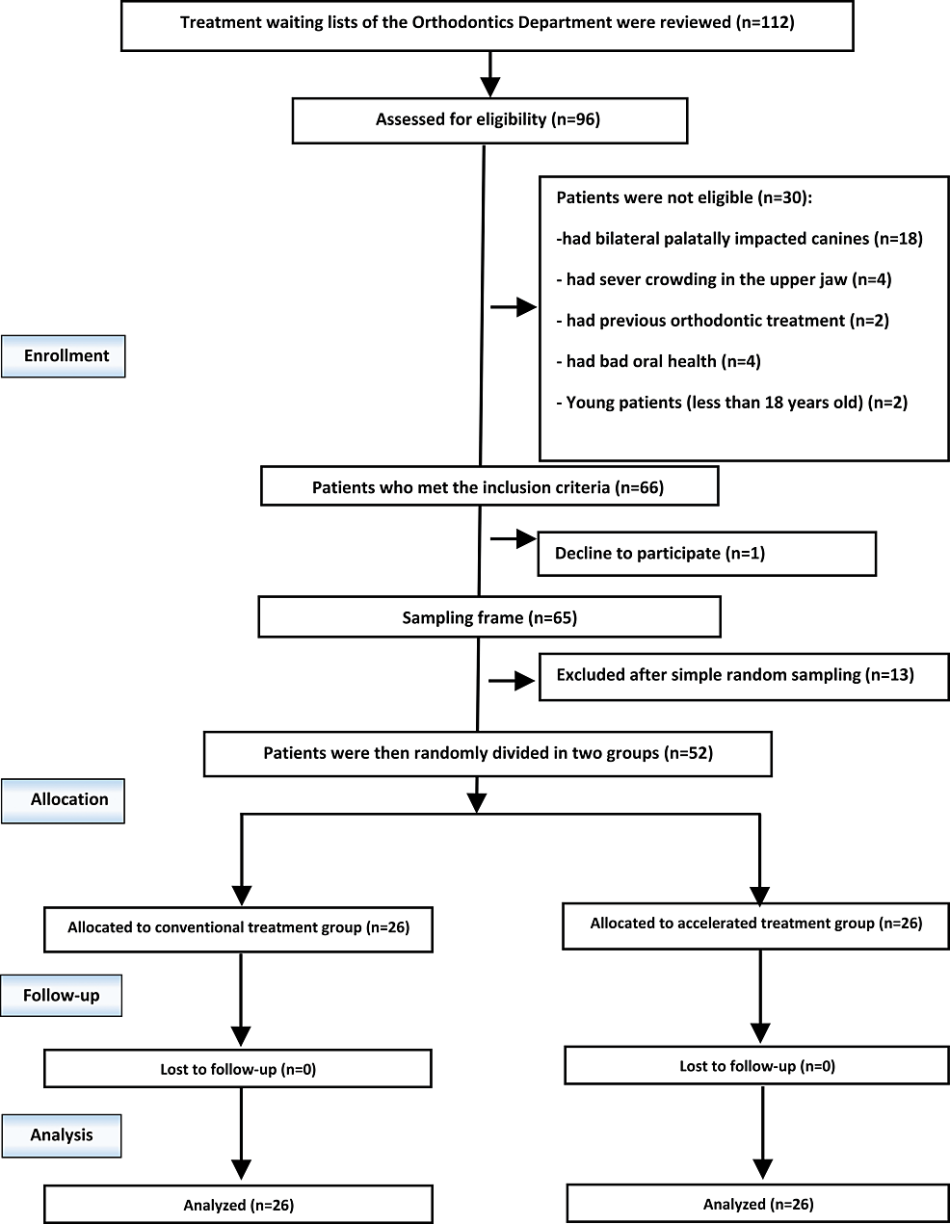
Consolidated Standards of Reporting Trials (CONSORT) flow diagram of patients' recruitment, follow-up, and entry to data analysis.

One hundred and twelve patients from treatment waiting lists of the Department of Orthodontics at the University of Damascus were screened. Sixty-six patients (18-30 years) who met the inclusion criteria were accepted. The treatment protocol was explained to all patients, and inquiries were answered. Sixty patients agreed to join the study, then 52 patients (13 males and 39 females) were randomly selected (Figure 1). All patients were given information sheets, and their informed consent was obtained. The inclusion criteria included the following: (1) adult patients (18-30 years old); (2) palatally or mid-alveolar unilateral impacted canine; (3) no previous orthodontic treatment; (4) healthy periodontal tissues and good oral health; (5) no consumption of any drug that may interfere with the tooth movement; (6) mild or no crowding in the upper jaw; and (7) no history of previous trauma to the maxillofacial region or surgical interventions. The exclusion criteria were the following: (1) any systemic diseases that would affect tooth movement; (2) antidepressant prevents oral surgery; (3) any congenital syndromes or cleft lip and palate cases; (4) bad oral health; and (5) previous orthodontic treatment.

Randomization, allocation concealment, and blinding

Using Minitab® software version 17, a list of random numbers was generated, and patients were randomly allocated according to this list using concealed allocation with an allocation ratio of 1:1. The selected 52 patients were distributed randomly into two groups: the conventional traction group (CT) comprising 26 patients and the corticotomy-assisted traction group (CAT) comprising 26 patients. Allocation concealment was done using sequentially numbered, sealed, opaque envelopes by one of the academic staff not involved in this study. For evident reasons, it was impossible to blind either study participants or practitioners to the interventions. However, before analyzing the data, the outcome evaluators were blinded and were unaware of the patient's intervention group.

Interventional groups

Orthodontic Procedures

**Figure 2:**
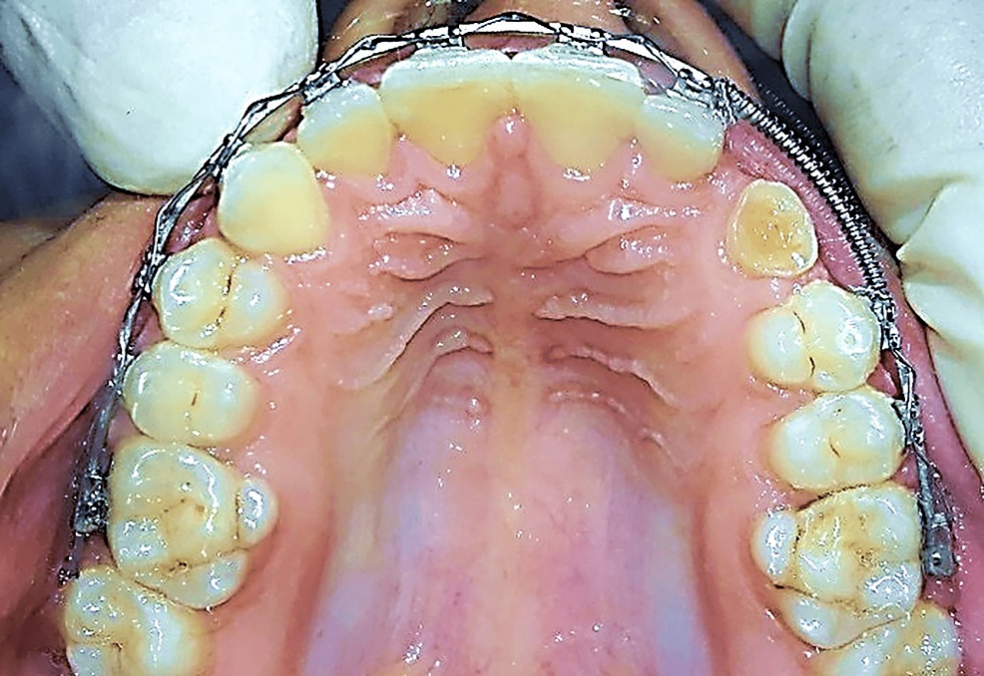
An open nickel-titanium (NiTi) coil spring was used for space opening before the beginning of canine traction.

**Figure 3:**
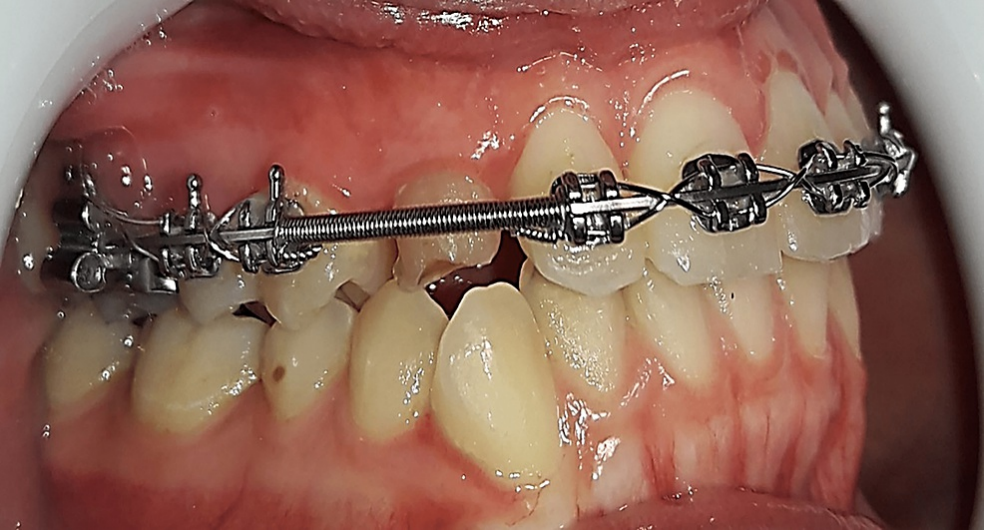
A stainless steel closed-coil spring was used to maintain the opened space before canine traction.

In both intervention groups, orthodontic treatment was performed with fixed appliances (McLaughlin, Bennett, and Trevisi prescription [MBT] 0.022-in slot, Votion™, Ortho Technology®, Florida, USA). Teeth leveling and alignment were performed using a series of elastic nickel-titanium (NiTi) wires: 0.012, 0.014, 0.016 x 0.022, and 0.017 x 0.025 NiTi wires (NT3®-SE NiTi Wire, American Orthodontics, Sheboygan, USA), and then a 0.019 x 0.025-inch stainless steel archwire was inserted (American Orthodontics, Sheboygan, WI, USA). Next, an appropriate space was opened for the impacted canine within the dental arch using a NiTi open-coil spring (Figure 2). This space was maintained using a stainless steel closed-coil spring (American Orthodontics, Sheboygan, WI, USA) (Figure 3).

Surgical Procedures

**Figure 4:**
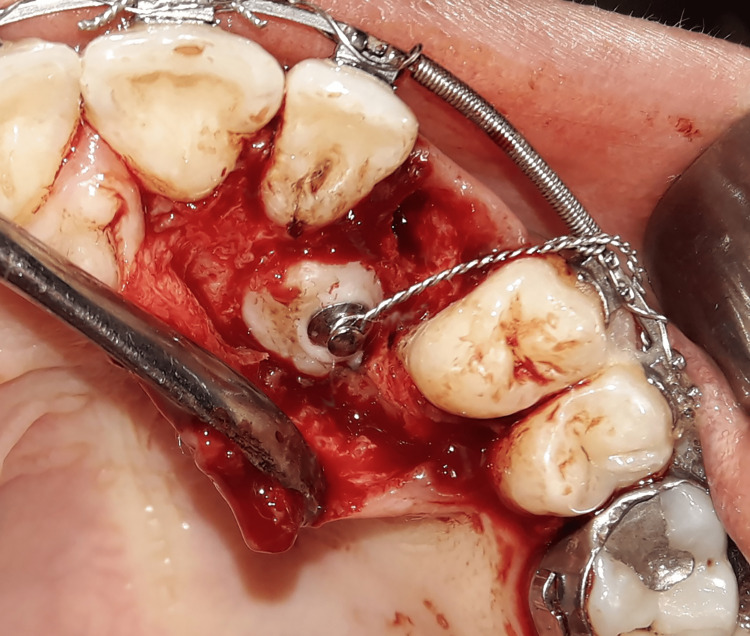
Bonding the stainless steel button on the exposed surface of the canine crown. A ligature wire was twisted around the button neck and extended outwards to be used for canine traction.

Conventional traction group (CT): After local anesthesia, the primary canine was extracted if present. Subsequently, a palatal full-thickness flap was raised, and the bone overlying the impacted canine was removed using a rounded surgical bur to expose an appropriate area of the impacted canine's crown. After obtaining a dry field using suction and surgical gauze, an eyelet attachment with a twisted ligature wire was bonded to the exposed impacted canine surface (Figure 4). The palatal mucosa was sutured back with the ligature wire extending through an incision in the palatal flap. Patients were given postoperative instructions as well as a prescription, including an antibiotic (clavulanic acid + amoxicillin, 1000 mg). If the patient required painkillers, he/she was first asked to fill out the questionnaire and was then allowed to take paracetamol 500 mg tablets. Patients were also asked to record the number of tablets taken.

**Figure 5:**
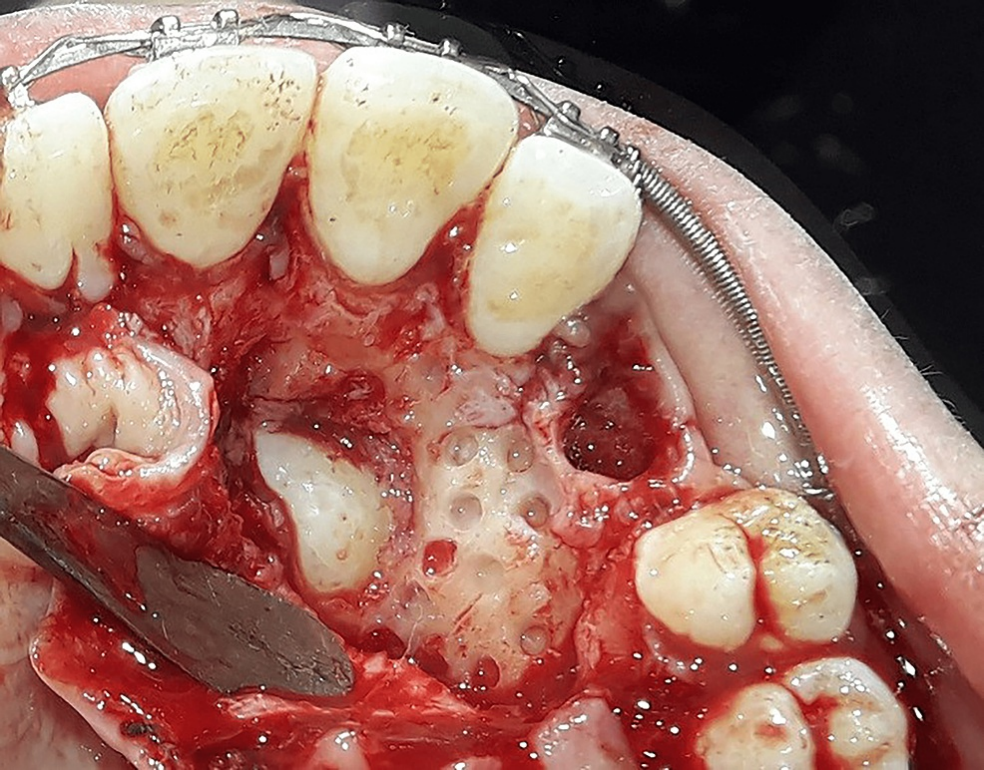
The corticotomy procedure: around the exposed canine crown, a series of circular holes (1-2 mm deep and spaced approximately 1.5 mm apart) were performed using a 1-mm round bur.

Corticomy-assisted traction group (CAT): The same surgical procedures were performed with an additional corticotomy to accelerate the traction movement. This procedure consists of several circular holes made using a 1 mm round bur about 1-2 mm in depth and 1 mm in diameter spaced about 1.5 mm apart in the mesial and distal bone surrounding the exposed canine crown as well as in the area to which the canine will be moved (Figure 5).

Outcome measures: pain, discomfort, and functional impairments

**Figure 6:**
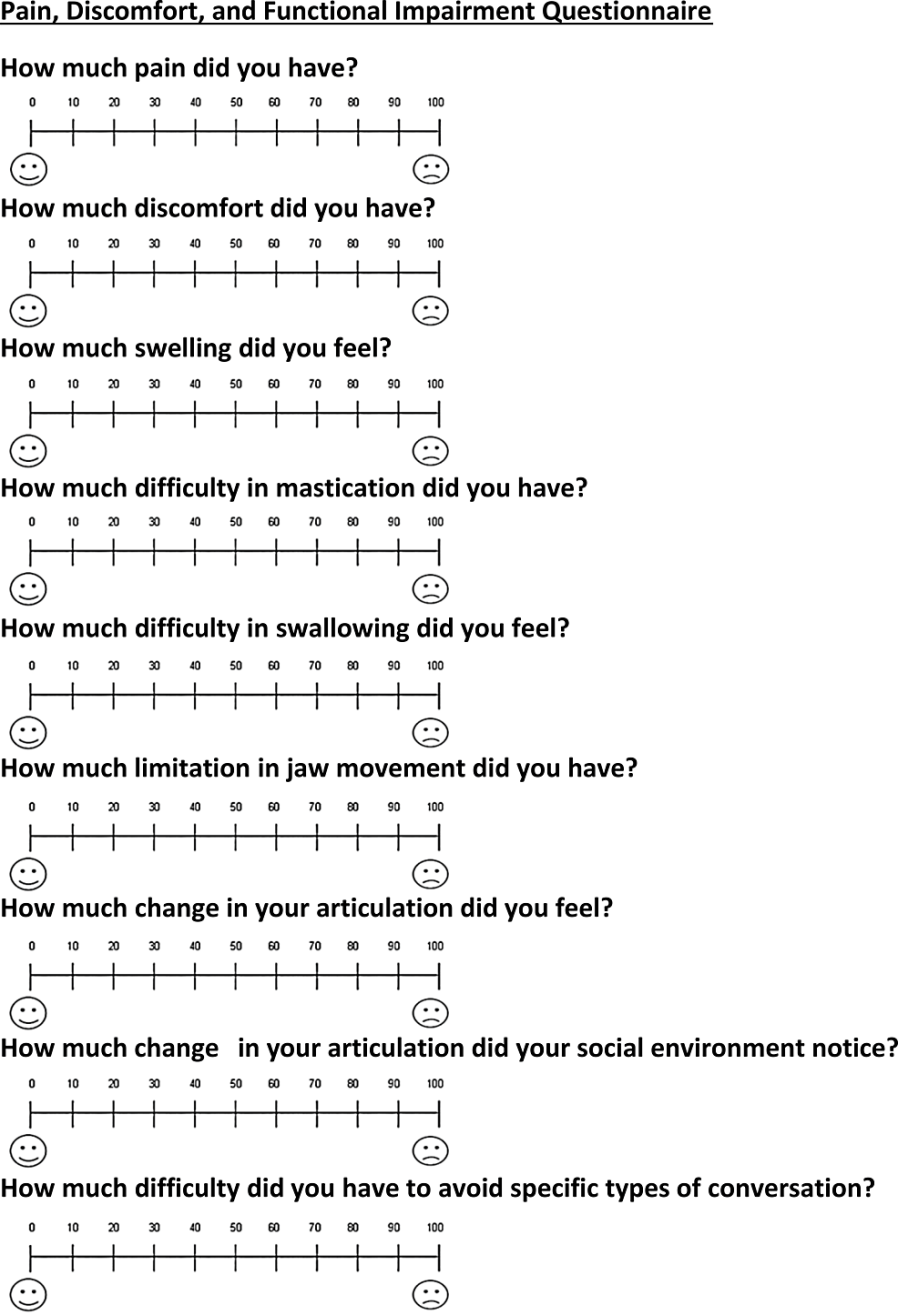
The questionnaire was administered to patients of both groups at one day (T1), four days (T2), seven days (T3), two weeks (T4), and four weeks (T5) following the surgical exposure.

**Figure 7:**
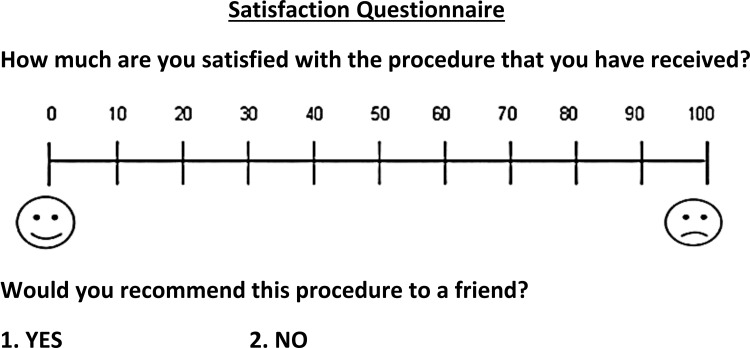
The questionnaire was administered only to patients in the accelerated traction group at 28 days following the surgical exposure.

One questionnaire was used in this study to obtain the outcomes related to pain, discomfort, and functional impairments during the first four weeks after surgical exposure of the impacted canines (Figure 6). Another questionnaire was used in the acceleration group only to assess the level of satisfaction among patients in this group (Figure 7). These questionnaires were used for the same objectives in previous studies [20,21] and were modified to conform to the current study. The eleven questions included in the questionnaires were about the levels of patient's perception of (1) pain, (2) discomfort, (3) swelling, (4) mastication difficulty, (5) swallowing difficulty, (6) jaw movement limitation, (7) patients' articulation changing, (8) patients articulation changing in their social environment, (9) avoidance of specific types of conversation, and (10) satisfaction with the accelerated surgical procedure. The last Yes/No question was: (11) would you recommend this accelerated surgical procedure to your friends?

Patients were asked to fill in the questionnaires at the following assessment times: 24 hours following the surgical intervention (T1), four days (T2), seven days (T3), two weeks (T4), and four weeks following the surgical exposure (T5). The patients determined the pain, discomfort, or functional problem levels by drawing a line on the VAS to express their feelings. The scale was a line with a 100-mm length. The left end of this line (i.e., score=0) indicated no pain, discomfort, or functional impairment, whereas the right end (i.e., score=100) indicated extreme sensation (i.e., maximum pain, discomfort, or functional impairment). In the acceleration group, patients' acceptance and satisfaction with the accelerated surgical intervention were assessed on a VAS at 28 days following surgical exposure; a score of zero represents the worst satisfaction from the surgical intervention, and a score of 100 represents the best satisfaction. In addition, at the last assessment time, patients were instructed to answer with Yes/No if he/she would recommend this accelerated surgical procedure to their friends.

Statistical analysis

All data analyses were performed using SPSS® Statistics program version 24.0 (IBM Corp., Armonk, NY, USA). Two-sample t-test and chi-square test were used to test the homogeneity between the two study groups according to gender and age, respectively. In addition, the Anderson-Darling Normality test was used to investigate the data distribution for each variable. These tests showed an abnormal distribution of data. Therefore, the Mann-Whitney U test was used to detect statistically significant differences between the two groups at each assessment time. Bonferroni's correction test was employed due to multiple comparisons, and the statistical significance was set at the 0.01 level.

## Results

Basic sample characteristics

The current study included 52 patients (11 males and 41 females). All patients had a palatally or mid-alveolar unilateral impacted canine. Twenty-six patients received conventional traction treatment (six males, 20 females, mean age: 20.37 ± 2.15 years), and twenty-six were treated with accelerated corticotomy-assisted treatment (five males, 21 females, mean age: 20.18 ± 2.18 years). The baseline characteristics of the included patients in each group are presented in Table 1. All patients' data were complete, as no withdrawals occurred at any assessment time point.

**Table 1:** Basic sample characteristics regarding gender and age. *Employing chi-square test;  **Employing two-sample t-test. CT: Conventional traction group; CAT: Corticotomy-assisted traction group; N: Number of patients; Min.: Minimum; Max.: Maximum.

Group	Gender	N (%)	P-value*	Mean age (SD)	Min. age	Max. age	P-value**
CT	Male	6 (23.07%)	0.987	20 (1.82)	18.00	23.00	0.718
	Female	20 (76.92%)		20.47 (2.17)	18.00	26.00	
CAT	Male	5 (19.23%)		20 ( 1.78)	18.00	23.00	
	Female	21 (80.76%)		20.22 (2.21)	18.00	25.00	
Both groups (n=52)	Male	11 (21.15%)		20 (1.80)	18.00	23.00	
	Female	41 (78.84%)		20.34 (2.19)	18.00	26.00	

Main findings

Descriptive statistics of patient-centered variables of questions 1 to 9 are shown in Table 2. The pain levels in the CT group were mild to moderate after 24 hours of the surgical exposure (36.80±25.45; T1), then decreased to become mild at T2, T3, and T4 (18.40±21.92, 8.80±15.36, 8.40± 15.19, respectively) and very mild at T5 (2.40±5.97). While the pain levels in the CAT group were mild to moderate after 24 hours (32.69±26.01; T1), then decreased to become mild at T2, T3, and T4 (16.54±15.22, 10.38±13.11, 6.15±9.83, respectively) and very mild at T5 (3.46±8.46). These levels were greater in the CT group at T1, T2, and T4, but the differences between the two groups were not statistically significant (p>0.01). In comparison, pain levels were greater in the CAT group at T3 and T5 without any significant differences between the two groups (p=0.379, 0.595, at T3 and T5, respectively).

**Table 2:** Descriptive statistics of patients' responses to questions 1-9 at the five assessment times for both groups using the visual analog scale and the results of significance testing. Mann–Whitney U test was used to detect statistically significant differences between the two groups at each assessment time, with Bonferroni's correction of the significance level (i.e., 0.05/5=0.01); P < 0.01 was considered statistically significant. CT: Conventional traction group; CAT: Corticotomy-assisted traction group; T1: 24 hours after the surgical exposure; T2: Four days after; T3: Seven days after; T4: 14 days after; T5: 28 days after the surgical exposure. Question 1: "How much pain did you have?"; Question 2: "How much discomfort did you have?"; Question 3: "How much swelling did you feel?"; Question 4: "How much difficulty in mastication did you have?"; Question 5: "How much difficulty in swallowing did you feel?"; Question 6: "How much limitation in jaw movement did you have?"; Question 7: "How much change in your articulation did you feel?"; Question 8: "How much change in your articulation did your social environment notice?"; and Question 9: "How much difficulty did you have to avoid specific types of conversation?"

Variables	Time	CT		CAT		CT vs. CAT			
		Mean (SD)	Median	Mean (SD)	Median	Mean Difference	95% CI for difference		P-value*
							Lower bound	Upper bound	
Q1: Pain	T1	36.80 (25.45)	30.00	32.69 (26.01)	30.00	4.11	-10.01	20.00	0.481
	T2	18.40 (21.92)	10.00	16.54 (15.22)	10.00	1.86	-10.01	10.00	0.823
	T3	8.80 (15.36)	0.00	10.38 (13.11)	10.00	-1.58	-10.00	0.00	0.379
	T4	8.40 (15.19)	0.00	6.15 (9.83)	0.00	2.25	0.00	0.00	0.894
	T5	2.40 (5.97)	0.00	3.46 (8.46)	0.00	-1.06	0.01	0.02	0.595
Q2: Discomfort	T1	44.00 (27.23)	50.00	35.00 (27.46)	30.00	9.00	-10.00	29.99	0.223
	T2	22.00 (25.00)	10.00	20.00 (16.25)	20.00	2.00	-10.00	10.00	0.735
	T3	14.40(18.73)	10.00	8.46 (14.05)	0.00	5.94	0.00	10.00	0.189
	T4	11.20 (16.91)	10.00	6.92 (10.87)	0.00	4.28	-0.01	10.00	0.486
	T5	3.20 (6.27)	0.00	4.23 (9.02)	0.00	-1.03	-0.00	0.00	0.823
Q3: Swelling	T1	36.40 (28.27)	30.00	27.69 (21.78)	25.00	8.71	-10.00	20.00	0.325
	T2	14.80 (15.58)	20.00	16.15 (17.68)	10.00	-1.35	-10.01	10.00	0.673
	T3	10.80 (15.25)	0.00	8.46 (13.77)	0.00	2.34	0.00	10.00	0.509
	T4	8.00 (13.23)	0.00	5.38 (9.05)	0.00	2.62	0.00	0.00	0.592
	T5	2.80 (8.43)	0.00	2.69 (7.24)	0.00	0.11	0.00	0.00	0.988
Q4: Mastication difficulties	T1	39.20 (29.43)	30.00	39.62 (29.32)	30.00	-0.42	-20.00	20.00	0.954
	T2	14.80 (21.63)	10.00	17.31 (24.42)	10.00	-2.51	-10.00	10.00	0.750
	T3	7.60 (12.00)	0.00	8.46 (15.67)	0.00	-0.86	0.00	0.00	0.830
	T4	4.80 (12.62)	0.00	6.15 (9.41)	0.00	-1.35	0.00	0.00	0.341
	T5	3.60 (12.54)	0.00	1.53 (4.64)	0.00	2.07	0.00	0.00	0.919
Q5: Swallowing difficulties	T1	26.40 (27.97)	20.00	15.00 (21.02)	10.00	11.4	0.01	20.00	0.112
	T2	11.60 (22.11)	0.00	13.85 (24.01)	0.00	-2.25	0.00	0.00	0.947
	T3	3.60 (10.36)	0.00	4.62 (10.29)	0.00	-1.02	0.00	0.00	0.379
	T4	4.00 (11.55)	0.00	1.53 (3.67)	0.00	2.47	0.00	0.00	0.869
	T5	0.80 (2.76)	0.00	0.38 (1.96)	0.00	0.42	0.00	0.00	0.547
Q6: Limitation in jaw movement	T1	22.00 (30.14)	20.00	18.85 (24.22)	10.00	3.15	-10.00	10.00	0.984
	T2	10.00 (20.62)	0.00	10.38 (19.90)	0.00	-0.38	0.01	0.00	0.973
	T3	4.00 (10.41)	0.00	7.31 (15.89)	0.00	-3.31	0.00	0.00	0.505
	T4	3.20 (9.88)	0.00	3.46 (9.36)	0.00	-0.26	0.00	0.00	0.764
	T5	2.00 (8.16)	0.00	1.15 (4.315)	0.00	0.846	0.00	0.00	0.967
Q7: Articulation changes	T1	30.80 (26.13)	30.00	17.31 (17.79)	20.00	13.49	0.00	20.00	0.046
	T2	10.40 (17.67)	0.00	10.38 (15.09)	5.00	0.02	-10.00	0.00	0.615
	T3	5.60 (13.87)	0.00	6.15 (12.99)	0.00	-0.55	0.00	0.00	0.505
	T4	2.80 (7.37)	0.00	3.85 (9.41)	0.00	-1.05	0.00	0.00	0.754
	T5	1.20 (4.39)	0.00	2.31 (5.14)	0.00	-1.11	0.00	0.00	0.276
Q8: Articulation changes in social environment	T1	29.60 (27.00)	30.00	16.54 (20.58)	10.00	13.06	0.01	30.00	0.077
	T2	10.00 (17.08)	0.00	7.31 (15.38)	0.00	2.69	0.00	0.00	0.565
	T3	5.60 (12.61)	0.00	4.62 (12.40)	0.00	0.98	0.00	0.00	0.979
	T4	3.20 (8.52)	0.00	3.85 (9.41)	0.00	-0.65	0.00	0.00	0.776
	T5	0.40 (2.00)	0.00	2.31 (5.14)	0.00	-1.91	0.00	0.00	0.095
Q9: Avoidance of specific types of conversation	T1	26.40 (27.52)	20.00	16.54 (22.26)	10.00	9.86	0.00	20.00	0.168
	T2	8.00 (13.23)	0.00	10.77 (19.17)	0.00	-2.77	0.00	0.00	0.786
	T3	5.20 (18.51)	0.00	7.69 (15.57)	0.00	-2.49	0.003	0.00	0.138
	T4	3.60 (10.75)	0.00	4.23 (7.58)	0.00	-0.63	0.00	0.00	0.372
	T5	0.80 (4.00)	0.00	2.31 (5.14)	0.00	-1.51	0.00	0.00	0.113

The discomfort levels in the CT group were mild to moderate after 24 hours (44.00±27.23; T1), then decreased to become mild at T2, T3, and T4 (22.00±25.00, 14.40±18.73, 11.20±16.91, respectively) and very mild after 28 days (3.20±6.27; T5). While the discomfort levels in the CAT group were mild to moderate after 24 hours (35.00±27.46; T1), then decreased to become mild at T2, T3, and T4 (20.00±16.25, 8.46±14.05, 6.92±10.87, respectively) and very mild at T5 (4.23±9.02). These levels were greater in the CT group at T1, T2, T3, and T4, but the differences between the two groups were not statistically significant (p > 0.01). While discomfort level was greater in the CAT group at T5, the difference between the two groups was nonsignificant (p=0.823).

Regarding swelling assessment, the levels were mild to moderate after 24 hours in the two groups, with no statistically significant difference (36.40±28.27and 27.69±21.78; in the CT and CAT groups, respectively; P=0.325). Then, these levels decreased to mild at T2, T3, T4, and T5 in both groups. These levels were greater in the CT group at T1, T3, T4, and T5, but the differences between the two groups were not statistically significant (p>0.01). In contrast, the swelling level was greater in the CAT group at T2 with no statistically significant difference (p=0.673).

The mastication difficulties levels in the CT group were mild to moderate at T1 and T2 (39.20±29.43, 14.80±21.63, respectively), then reduced to become mild at T3 (7.60±12.00) and very mild at T4 and T5 (4.80±12.62, 3.60±12.54, respectively). While they were mild to moderate in the CAT group at T1 and T2 (39.62±29.32, 17.31±24.42, respectively), then reduced to become mild at T3 and T4 (8.46±15.67, 6.15±9.41, respectively) and very mild at T5 (1.53±4.64). These levels were greater in the CAT group at T1, T2, T3, and T4, but it was greater in the CT group at T5, with no statistically significant difference between the two groups (p>0.01).

The difficulties of swallowing levels were mild to moderate in the CT group at T1 (26.40±27.97), then reduced to become mild at T2 (11.60 ±22.11) and very mild at T3 and T4 (3.60±10.36, 4.00±11.5, respectively). In comparison, they were mild in the CAT group at T1 and T2 (15.00±21.02, 13.85±24.01, respectively) and very mild at T3 and T4 (4.62±10.29, 1.53±3.67, respectively). These levels were greater in the CT group at T1 and T4, but it was greater in the CAT group at T2 and T3, with no statistically significant difference between the two groups (p>0.01). The difficulties in swallowing were almost non-existent in the two groups at T5, with no significant difference between the two groups (0.80±2.76, 0.38±1.96, in the CT and CAT groups, respectively; P=0.547).

The limitation in jaws movement levels was mild to moderate in the CT group at T1 and T2 (22.00±30.14, 10.00±20.62, respectively), then reduced to become very mild at the other following assessment times (4.00±10.41, 3.20±9.88, 2.00±8.16; at T3, T4, and T5, respectively). In comparison, they were mild in the CAT group in the first week (18.85±24.22, 10.38±19.90, 7.31±15.89; at T1, T2, and T3, respectively) and very mild at the other following assessment times (3.46±9.36, 1.15±4.31; at T4 and T5, respectively). These levels were greater in the CT group at T1 and T2, but it was greater in the CAT group at T3, T4, and T5, with no statistically significant difference between the two groups (p>0.01).

Regarding the subjective assessment of speech, the articulation changes levels were mild to moderate in the CT group on the first postoperative day (30.80±26.13), then reduced to become mild at T2 and T3 (10.40±17.67, 5.60±13.87, respectively) and very mild at T4 and T5 (2.80±7.37, 1.20±4.39, respectively). In comparison, they were mild in the CAT group in the first week (17.31±17.79, 10.38±15.09, 6.15±12.99; at T1, T2, and T3, respectively), then reduced to become very mild at T4 and T5 (3.85±9.41, 2.31±5.14, respectively). The differences between the two groups were statistically insignificant at all assessment times (p>0.01).

When the surrounding people's observation was evaluated, the articulation changes levels were mild to moderate in the CT group at T1 (29.60±27.00), then reduced to become mild at T2 and T3 (10.00±17.08, 5.60±12.61, respectively) and very mild at T4 (3.20±8.52), while after four weeks the patients rated that their articulation was almost unrestricted (0.40±2.00; T5). In the CAT group, these levels were mild at T1 and T2 (16.54±20.58, 7.31±15.38, respectively), then reduced to become very mild at the other following assessment times (4.62±12.40, 3.85±9.41, 2.31±5.14; at T3, T4, and T5, respectively). The differences between the two groups were statistically insignificant at all assessment times (p>0.01).

Regarding conversation avoidance, patients in the CT group had mild to moderate levels of avoidance of some types of conversations at T1 (26.40±27.52), then these levels reduced to become mild at T2 and T3 (8.00±13.23, 5.20±18.51, respectively) and very mild at T4 (3.60±10.75). While after four weeks, the patients reported that they almost did not avoid any type of conversation (mean value: 0.80±4.00; T5). In the CAT group, these levels of conversation avoidance were mild in the first week (16.54±22.26, 10.77±19.17, 7.69±15.57; at T1, T2, and T3, respectively), then reduced to become very mild at T4 and T5 (4.23±7.58, 2.31±5.14, respectively). These levels were greater in the CT group only at T1, with no statistically significant difference between the two groups (p>0.01).

After four weeks of the accelerated surgical intervention, patients' satisfaction with this intervention in the CAT group was high (91.5±9.5), and 92.3% of patients recommended this intervention to their friends.

## Discussion

The current study seems to be the first randomized clinical trial to investigate the levels of pain, discomfort, and functional impairments associated with the conventional method of PICs traction compared to the accelerated traction method using the minimally invasive corticotomy-assisted technique. The VAS scale was used in this study because it has many proven advantages, as it is a practical, simple, and effective method for evaluating these variables [14,27]. In addition, it can also estimate changes in sensation intensity over time [14].

The current study showed no significant differences in the pain levels between the CAT group in comparison with the CT group at all assessment times. The pain levels were mild to moderate on the first postoperative day, and only 11.5% of patients in each study group had severe pain. These results were in agreement with those of the closed surgical groups in the prospective studies of Björksved M et al. and Gharaibeh TM and Al-Nimri KS [18,19], who used CT methods and in disagreement with the results of Chaushu S et al. [21], who reported a high level of pain (score 8-10 of 10) in both closed and open-surgical methods on the first postoperative day by 27.6% of the sample. The difference can be attributed to the inclusion of palatal and buccal impactions in their study, whereas in the current work, only palatal or mid-alveolar impaction was included. In addition, the type of surgical exposure used in that study varied between open and closed approaches, whereas in the current study, only closed traction was employed.

Since there are no studies that have evaluated pain levels in patients undergoing accelerated traction of PICs, a direct comparison of the current results with other studies is not possible. However, compared with other trials evaluating surgically assisted accelerated orthodontics, the current findings were inconsistent with most of those studies. For example, Kundi I et al. and Babanouri N et al. evaluated the acceleration of canine retraction movement using the micro-osteoperforations (MOPs) technique and found significant higher pain levels in the surgical group than in the CT group within 24 hours postoperatively [28,29]. Our results were also inconsistent with the findings by Shahrin AA et al., who evaluated the acceleration of leveling and alignment in the initial orthodontic stage using MOPs. These differences in the pain levels on the first postoperative day can be attributed to the differences in the study's design of these trials from the current study, the location of the corticotomy procedure, and the difference in sample characteristics such as age, gender, sample size, and type of malocclusion. In addition, using a split-mouth design in some studies can affect the patient's ability to assess pain severity.

The pain levels decreased similarly in the two study groups at the following assessment times and became mild during the first and second weeks, then very mild and almost non-existent after four weeks, with no significant differences between the two groups. Our results related to the first postoperative week were consistent with those reported by Björksved M et al., Gharaibeh TM and Al-Nimri KS [18,19]. At the same time, there were no data about the pain levels after day 7 in these two studies. In addition, Parkin NA et al. [20] reported that mild pain lasted for several days after the surgical exposure in 69% of the closed-eruption group and more than one week in 10% of this group. However, their data were collected only on the 10th day postoperatively, i.e., missing the most important events in the immediate postoperative stage. However, the current results were inconsistent with the results of Chaushu S et al. [21], who found that the recovery time was significantly shorter in the closed-eruption group as the pain level and the need for analgesics decreased after the second postoperative day.

While the current findings showed that the pain levels were mild to moderate on the first and fourth postoperative days, some trials that used the MOP technique [28,30] indicated that the pain levels were high on the second and third days after the surgical intervention. These differences can be attributed to the fact that the holes in the current study were about 1-2 mm deep and 1 mm in diameter, whereas, in those studies, they were 3-4 mm deep and 1.5 mm in diameter. Moreover, in most cases of the current study, it was impossible to perform more than 5-6 holes. This was less than in the previous two studies, where the number of holes ranged from 6 to 8 in all cases. Therefore, the method used in this study results in less bone injury than the MOP technique and therefore causes lower pain levels.

In the current study, the discomfort levels were mild to moderate on the first postoperative day in both study groups. These levels then decreased to mild during the first and second weeks. Subsequently, discomfort levels became mild and almost non-existent on the 28th day after the surgical exposure. No significant differences were found between the two groups at all assessment times. Similar results were found by Parkin NA et al. [20], who concluded that in most patients, the discomfort was of short duration and subsided after a few days.

Chaushu S et al., in their clinical trial that evaluated the open versus closed-eruption techniques, reported that the exposure of PICs by the closed technique produced fewer discomfort levels. Our results were in line with this finding and may be explained by the anatomical structure of the palatal side, as the palatally mucosa is fully attached, and the surgical flap is located in a less mobile oral mucosa compared to the buccal side [21]. Unfortunately, the results of discomfort levels related to the acceleration procedure in the current study cannot be compared with the results of others due to the absence of similar studies.

Regarding the swelling levels, the results of the current work were in disagreement with the findings of Chaushu G et al. [31], who reported severe swelling in 34.4% of the study group on the first postoperative day. The proportion of patients who suffered from severe swelling decreased to 3.4% on the fourth day, and this complaint completely disappeared after one week. In addition, the author indicated that the recovery of swelling in patients with palatally impaction teeth was faster than in buccal impaction and disappeared on the second postoperative day [31]. These differences can be attributed to the type of orthodontic/surgical interventions in the current study from that retrospective study, which included different tooth impactions. However, the results of the current study were in agreement with the study of Alkebsi A et al. [32], who evaluated the effect of micro-osteoperforations (MOPs) on the rate of tooth movement during canine retraction and reported that the feeling of swelling on the side of the MOPs was mild at all assessment times after the acceleration procedure.

During the five assessment times, no significant differences were found in the mastication difficulties levels between the two study groups. Therefore, the results of the current study were inconsistent with those of the two studies by Chaushu S et al. and Chaushu G et al. [21,31] but consistent with the findings of Björksved M et al. [18]. Chaushu S et al. and Chaushu G et al. reported that on the first postoperative day, difficulty in eating was the most frequently reported problem (65.5% of patients), followed by the inability to enjoy regular food (31.0%). Then the improvement in oral function in these studies was evident by day four postoperatively [21,31]. However, oral function evaluations in these two studies were reported as the proportions of patients with functional impairments, while in the current study, the assessments were reported using mean scores. Difficulty in swallowing was mild at almost all assessment times in both groups, with no significant differences. The recovery time required four days to reach minimal levels. This can be attributed to the closed traction method in both groups, where the palatal flap was sutured back, and the orthodontic attachments bonded to the impacted canine were covered. Therefore, it did not interfere with tongue movement. These results were consistent with those of Chaushu S et al. [21], who found that only 3% of patients in the closed traction group had severe swallowing difficulty in the three days following the surgical exposure, with an average recovery time of one day for patients with palatal impactions.

Limitation of jaw movement was reported as mild to moderate in the two study groups immediately after the surgical exposure, i.e., days one and four, without any significant differences. Then the level of jaw movement limitation was decreased to very mild after the first week. These findings were in line with the results of two studies by Chaushu S et al. and Chaushu G et al. [21,31], who reported that only 20% of patients in the closed traction group had severe difficulty opening their mouths in the two days following the surgical exposure with an average recovery time of two days.

In the present study, the speech changes assessed by patients or their social environment were low to moderate in both study groups on the first postoperative day without any significant differences. Similar results were found regarding patients attempting to avoid specific types of conversations. These articulation changes can be explained by the fact that patients tried to keep the tongue out of contact with the area of surgical intervention, particularly on the first postoperative day. The low level of speech distortion can be attributed to the low thickness of orthodontic attachments bonded to the impacted canines that were covered by the sutured palatal flap; therefore, the movement of the tongue during the speech was not significantly restricted. However, the level of speech impairment in both study groups decreased to low levels after four days. These results were consistent with the results of the two studies by Chaushu S et al. and Chaushu G et al. [21,31], who found that the recovery time regarding the severe difficulty in the speech was one day after the surgical exposure in the closed traction group, and the improvement in the speech was evident by the seventh day postoperatively.

Limitations

Assessing the pain, discomfort, and functional impairment levels was not performed daily during the first postoperative week. This is one of the limitations of this trial. Second, only PICs were included in this study, and other types of impaction were not considered. Third, the follow-up duration was only four weeks, while the traction force was applied every 2 to 3 weeks for 5 to 14 months. This may lead to a repeated sensation of pain and discomfort at each activation session, which was not evaluated in the current study. Additionally, this study did not evaluate gender and age differences in the perception of pain, discomfort, and functional impairments, which require a larger sample size.

Generalizability

The generalizability of the current study results may be limited, as it included patients with a specific type of canine impaction with a specific age group treated with a specific orthodontic/surgical technique in only one orthodontic center.

## Conclusions

Both the conventional and corticotomy-assisted methods caused mild to moderate levels of pain, discomfort, and functional impairments on the first postoperative day, with no significant differences at all assessment times. These effects gradually decreased during the first and second weeks and almost completely disappeared after four weeks of surgical exposure. Patient satisfaction with the corticotomy procedure performed was high, with 92.3% of patients agreeing to recommend this acceleratory intervention to a friend. The results of the current study may suggest that the corticotomy-assisted method used to accelerate the PIC's traction movement is a comfortable procedure and does not cause a significant increase in the levels of pain and discomfort when compared to the conventional method.
